# Supplementary material for: The effects of genital myiasis on the diversity of the vaginal microbiota in female Bactrian camels
Source: BMC Vet Res. 2022 Mar 5;18:87. doi: 10.1186/s12917-022-03189-5 (PMC8897907; doi:10.1186/s12917-022-03189-5)
Supplement: Supplementary file 5 — Additional file 5. [file 12917_2022_3189_MOESM5_ESM.zip › MPL201709200_16s_yy/Treat1/css/js_css/pdfmake-master/examples/pdfs/named_styles_with_overrides.pdf]

This paragraph uses header style and extends the alignment property

This paragraph uses header style and overrides bold value setting it back to false.

Header style in this example sets alignment to justify, so this paragraph should be rendered

Lorem ipsum dolor sit amet, consectetur adipisicing elit. Malit profecta versatur nomine ocurreret multavit, officiis viveremus aeternum superstitio suspicor alia nostram, quando nostros congressus susceperant concederetur leguntur iam, vigiliae democritea tantopere causae, atilii plerumque ipsas potitur pertineant multis rem quaeri pro, legendum didicisse credere ex maluisset per videtis. Cur discordans praetereat aliae ruinae dirigentur orestem eodem, praetermittenda divinum. Collegisti, deteriora malint loquuntur officii cotidie finitas referri doleamus ambigua acute. Adhaesiones ratione beate arbitraretur detractis perdiscere, constituant hostis polyaeno. Diu concederetur.
